# Depressive Symptom Clusters and Neuropsychological Performance in Mild Alzheimer's and Cognitively Normal Elderly

**DOI:** 10.1155/2011/396958

**Published:** 2011-08-29

**Authors:** James R. Hall, Sid E. O'Bryant, Leigh A. Johnson, Robert C. Barber

**Affiliations:** ^1^Institute of Aging and Alzheimer's Disease Research, University of North Texas Health Sciences Center, Fort Worth, TX 76107, USA; ^2^Department of Psychiatry, University of North Texas Health Sciences Center, Fort Worth, TX 76107, USA; ^3^F. Marie Hall Institute for Rural and Community Health, Texas Tech University Health Sciences Center, Lubbock, TX 79415, USA; ^4^Department of Neurology, Texas Tech University Health Sciences Center, Lubbock, TX 79415, USA; ^5^Laura W. Bush Institute for Women's Health, Texas Tech University Health Sciences Center, Amarillo, TX 79106, USA; ^6^Department of Family and Community Medicine, Texas Tech University Health Science Center, Lubbock, TX 79415, USA; ^7^Department of Pharmacology and Neuroscience, University of North Texas Health Sciences Center, Fort Worth, TX 76107, USA

## Abstract

*Objectives*. Determine the relationship between depressive symptom clusters and neuropsychological test performance in an elderly cohort of cognitively normal controls and mild Alzheimer's disease (AD). *Design*. Cross-sectional analysis. *Setting*. Four health science centers in Texas. *Participants*. 628 elderly individuals (272 diagnosed with mild AD and 356 controls) from ongoing longitudinal study of Alzheimer's disease. *Measurements*. Standard battery of neuropsychological tests and the 30-item Geriatric Depression Scale with regressions model generated on GDS-30 subscale scores (dysphoria, apathy, meaninglessness and cognitive impairment) as predictors and neuropsychological tests as outcome variables. Follow-up analyses by gender were conducted. *Results*. For AD, all symptom clusters were related to specific neurocognitive domains; among controls apathy and cognitive impairment were significantly related to neuropsychological functioning. The relationship between performance and symptom clusters was significantly different for males and females in each group. *Conclusion*. Findings suggest the need to examine disease status and gender when considering the impact of depressive symptoms on cognition.

## 1. Introduction

A number of studies have reported an association between depressive symptoms and cognitive dysfunction in the elderly [1–3] with scores on depression scales being significantly related to specific neurocognitive domains of visuospatial skills, executive functioning, psychomotor speed, as well as memory [4–6]. Depressed but cognitively normal elderly subjects often differ in their performance on neuropsychological tests from Alzheimer's disease (AD) patients [7]. Specific patterns of performance on neuropsychological tests have been found to differentiate depression from AD [8]. However, little research has examined the potential impact of specific depressive symptoms or symptom clusters on detailed neuropsychological functioning.

 The majority of research examining the neurocognitive consequences of depression has utilized total scores on depression scales, groupings based on cutoff scores on a rating scale, or diagnosis of a depressive disorder based on a variety of criteria (e.g., DSM-IV-TR). However, symptom clusters as well as depressive endophenotypes have been well documented to have clinical relevance. Furthermore, the treatment of depression as a unitary concept obscures the link between specific areas of cognitive functioning and these depressive symptom clusters. The limited research that has been conducted with depressive symptom clusters has shown that specific symptoms differentially relate to performance on neuropsychological tests. Janzing and colleagues [9] studying a sample of 60 demented subjects used a principal components analysis to derive a motivation factor and a mood factor from the DSM-III criteria for depression. They found that the motivation factor was related to performance on measures of verbal fluency while the mood factor and general depressive symptoms did not relate to performance on any neuropsychological test. Castro-Costa and colleagues [10] in a study of a large European sample of elders from ten countries in the SHARE sample found that the motivation factor score of the EURO-D depressive screening scale was significantly related to poorer performance on a measure of verbal fluency but not to verbal memory. The affective suffering factor was not significantly related to any neuropsychological test scores. O'Bryant and colleagues [11] examined data from 184 participants of Project FRONTIER, an ongoing epidemiological study of rural health, and found that depressive symptom clusters were specifically associated with neuropsychological domains. In that study, depressive feelings of dysphoria, meaninglessness, and cognitive impairment were significantly associated with memory, language, and attention domains; however, the pattern of significance varied when the sample was split by gender and ethnicity. As these studies show, subtypes of depressive symptoms are differentially related to specific neurocognitive domains. However, this question has not been investigated in a sample of elderly patients with or without Alzheimer's disease.

The present study investigated the relationship between the four clusters of depressive symptoms of the GDS identified by Hall and Davis [12] and performance on a standard battery of neuropsychological tests in a cohort of cognitively intact elderly and elderly diagnosed with mild Alzheimer's disease (AD). It was hypothesized that depressive symptoms and neuropsychological testing would interact differentially among cognitively normal elderly compared to elderly subjects with AD. Previous research [11] on the subscales has shown significant differences between males and females in their endorsement patterns and impact on neuropsychological test scores in a nondemented ethnically diverse sample. Therefore, it was also hypothesized that males and females in both the cognitively normal group and AD group would exhibit distinct patterns of relationships between symptom clusters and neuropsychological functioning.

## 2. Methods

### 2.1. Participants 

Participants included 628 individuals (272 diagnosed with probable AD and 356 cognitively intact) enrolled in the longitudinal research cohort of the Texas Alzheimer's Research Consortium (TARC), a well-characterized representative cohort of AD and normal controls assessed annually. The methodology of the TARC project has been described in detail elsewhere [13]. Briefly, each participant undergoes an annual evaluation that includes a medical examination, interview, neuropsychological testing, and blood draw. AD patients met consensus-based diagnosis for probable AD based on NINCDS-ADRDA criteria [14] and scored 0.5 or 1.0 on the CDR global score. Controls performed within normal limits on psychometric assessment and were assigned a CDR global score of 0.0. The breakdown of the CDR global scores was as follows: 0 = 356 (controls), 0.5 = 71 (AD), and 1.0 = 201 (AD). The vast majority of participants were Caucasian (93%). The characteristics of the participants are presented in Table 1. The TARC project received Institutional Review Board approval and all participants and/or caregivers signed written informed consent documents.

### 2.2. Measures

The TARC neuropsychology core battery consisted of commonly utilized instruments including Wechsler digit span, Trail Making Test, Wechsler Logical Memory, Wechsler Visual Reproduction, Boston Naming Test (30- and 60-item versions), verbal fluency (FAS), the Geriatric Depression Scale (GDS-30), and the Clinical Dementia Rating scale (CDR). In order to equate scores from digit span and story memory scales across test versions, all raw scores were converted to scale scores based on previously published normative data. For the Boston Naming Test, the authors recently conducted an independent study that demonstrated the psychometric properties of an estimated 60-item BNT score that is calculated from 30-item versions [15]. Scale scores adjusted for age and education were utilized as dependent variables in analyses.

**Geriatric Depression Scale (GDS) Subscales**

The Geriatric Depression Scale [16] was the first depression scale primarily used as a screening instrument designed for older populations and has been used widely in clinical settings and research with the elderly. However, the GDS was not specifically designed for use with cognitively impaired elderly. The vast majority of studies using the GDS have employed total score or a clinical cutoff. A recent factor study [12] on older individuals with cognitive impairment identified four factors of the GDS which the authors describe as (1) dysphoria factor, (2) meaninglessness factor, (3) apathy factor, and (4) Cognitive Impairment factor. The Dysphoria factor contains 11 items primarily associated with a sad mood. The Meaninglessness factor consists of seven items that reflect an appraisal of the meaning (or lack thereof) in one's life. The apathy factor is made up of six items that reflect a lack of motivation or initiative. The cognitive impairment factor consists of six items that reflect difficulty and concern with cognitive processes. The GDS subscale scores were utilized as predictor variables. Although these subscales were developed using a mixed sample of cognitive disorders, they have been found to be useful when investigating the relationship between neuropsychological test performance and depression in nondemented samples [11].

#### 2.2.1. Analysis

Stepwise linear regression models were generated for controls and AD subjects with GDS subscale scores entered as predictor variables and the neuropsychological test scores entered as outcome variables, with age, gender, and education entered as covariates. Follow-up regression models were generated for males and females in each group. Significance for analyses was set at *P* < .05. Adjusted scale scores were used for Digit Span, Logical Memory I and II, Visual Reproduction I & II, Trails A & B, FAS, and Boston Naming.

## 3. Results

The controls were significantly younger and more educated than the AD group (*P* ≤ .001). No significant differences were found between males and females within either group on age or education (*P* ≥ .05). Multivariate analysis of variance found no significant differences between AD males and females on any of the subscales (*P* ≥ .05). There were no significant differences between males and females on any of the subscales for the control group (*P* ≥ .05; see Table 1 for means and standard deviations). 

Table 2 presents the outcomes of the regression analyses for the control group. As shown, none of the depressive symptom subscales were significantly related to verbal fluency, Boston Naming, or Digit Span. However, the cognitive Impairment subscale was significantly associated with poorer scores on Trails B as well as immediate and delayed memory for verbal and visual information. The apathy subscale was negatively associated with scores on Trails A. 

When examined by gender, there were no significant findings for control males. However, for female Controls at least one of the GDS subscales was a significant negative predictor for all the neuropsychological measures except the Boston Naming Test and Digit Span (Table 2). Cognitive impairment was the most robust predictor of poor performance being significantly negatively related to Trails B, Logical Memory I, Logical Memory II, Visual Reproduction I, and Visual Reproduction II. The meaninglessness subscale significantly negatively predicted verbal fluency while apathy negatively predicted Trails A scores. 

Table 3 presents the stepwise regression models generated for the AD sample. None of the subscales were significantly related to performance on Boston Naming or WMS Visual Reproduction I and II. Dysphoria subscale scores were significantly related to poorer scores on Verbal Fluency, Trails A, Trails B, and Digit Span for the total sample of AD patients. Apathy was significantly related to poorer immediate and delayed verbal memory (WMS Logical Memory I and II) while the meaninglessness subscale scores were negatively related to delayed verbal recall (WMS Logical Memory II). The cognitive impairment subscale was significantly and positively related to measures of delayed recall of verbal information. Analyses broken down by gender (Table 3) again revealed a different picture than was observed from analyses conducted with the total sample. Among women diagnosed with AD, scores on Verbal Fluency, Logical Memory II, and Visual Reproduction I were significantly negatively related to the Apathy subscale. Trails A and B were both affected negatively by the dysphoria subscale. For AD males, Trails B was negatively associated with the cognitive impairment subscale while Digit Span was negatively associated with dysphoria. No other neuropsychological tests had a significant relationship to depressive symptom clusters for AD males. 

## 4. Discussion

The current findings suggest that the link between depression and cognitive functioning is quite complex. These results demonstrate that specific clusters of depressive symptoms and disease status, as well as gender can differentially impact neuropsychological domains. These findings point to the need to specifically examine disease status and gender when considering the effect of depressive symptoms on neuropsychological functioning as these relationships are obscured in the overall models when simply covarying for these factors. 

This is further illustrated by our examination of the relationship between symptom clusters and neuropsychological performance by case status (Alzheimer's disease or normal controls). Among patients diagnosed with AD, the GDS-30 subfactors of dysphoria and apathy were the factors most consistently associated neuropsychological testing. Elevations in dysphoria and apathy were related to poorer scores in attention and executive functioning and verbal fluency and memory, respectively. It is interesting to note that among AD cases scores on the cognitive impairment subscale were significantly associated with better scores on delayed memory (visual and verbal) among the total sample. However, when the sample was split by gender, a different picture was observed as the vast majority of the findings were held only for women, which is consistent with our earlier findings [11]. 

The cognitively normal controls presented a very different pattern of relationships between subfactors and cognition. In this sample, the subfactor of cognitive impairment was, by far, the most robust predictor of neuropsychological test performance. However, as with the AD sample, a different profile was seen when broken down by gender such that the link between depressive subfactors and cognitive status only held for women. 

These findings point to the need to directly examine cohorts by demographic factors rather than covarying for them when considering the complex impact depression has on health status. Our findings suggest that the link between depression and neuropsychological functioning is most relevant for women, particularly among those without a neurodegenerative disorder. Rosenberg and colleagues [17] in a longitudinal study of cognitively healthy older women used total GDS score and found a strong relationship between number of depressive symptoms and decline in a number of cognitive domains. In our study we found that the specific depressive symptom cluster related to concern with cognitive status was significantly related to poorer cognitive performance in cognitively normal older women. These findings directly contradict the old adage “if they present with complaints of cognitive dysfunction they are likely depressed” and suggest that such a notion may potentially prevent women who have real cognitive disturbances from receiving appropriate attention. 

Our findings also demonstrate that the meaning of depressive symptoms changes once an individual has been diagnosed with a neurodegenerative dementia syndrome, such as Alzheimer's disease. Among Alzheimer's disease cases, it is feelings of apathy and dysphoria that are most important. These findings are not surprising given the wealth of the literature documenting social withdrawal and isolation as an early sign (or consequence) of AD. However, given that social withdrawal is also a symptom of depression, it is important that elders presenting with these specific symptoms of depression be referred for a comprehensive dementia examination so that treatment can be implemented early in the course of the disease when treatments are most efficacious.

The generalizability of our findings is limited by the nature of the sample. The study is cross-sectional and the TARC cohort at this time is predominately Caucasian, relatively well-educated, and, for the most part, urban. Our sample was limited to mild AD which does not allow us to evaluate the affect of these symptoms as the disease progresses. Additionally individuals with high levels of depression initially or with a diagnosis of major depression are excluded from the cohort. It is therefore difficult to determine the impact of level of depression. As can be seen from Table 1, the overall level of depressive symptoms is relatively low for both AD and controls. Even with the low number of depressive symptoms endorsed there is still a differential impact of symptom clusters on neuropsychological domains. The subscales used in this research were originally developed through factor analysis of a sample of individuals with a variety of cognitive disorders. The application of these scales to a cognitively normal sample may be a limitation although previous research on cognitively intact elderly [11] using the subscales has shown their utility in describing the relationship of depressive symptom clusters to neuropsychological performance. Additionally the role that education may play in moderating the relationship between depressive symptoms and neuropsychological performance was not directly investigated. It is likely that the effect of education was controlled to at least some extent through the use of education-adjusted neuropsychological scales scores and the finding that education did not significantly impact any of the relationships reported. 

A potential implication for the current findings is on rate of progression. Given the current findings of a differential link between specific depressive symptoms and cognitive functioning between AD cases and controls, it is possible that the depressive symptoms most important for conveying risk of cognitive decline over time will vary. This is of critical importance as the current investigators are unaware of any prior work examining if specific symptoms (or symptom clusters) of depression are most important in predicting decline over time or if these “depressive risk symptoms” differ by case or controls status. That is, are specific symptoms of depression related to the risk for developing Alzheimer's disease in the future and are a different set of depressive symptoms important for rate of decline once the disease has become clinically manifest? 

 The current findings may suggest a better understanding of the neurobiological consequences of AD. While there has been a wealth of literature documenting alterations in inflammatory and other neurochemical pathways in both AD and depression, no prior research has examined if the specific symptoms of depression are differentially related to these pathways. If specific symptoms of depression (i.e., apathy and dysphoria) are specifically related to modifiable pathways (e.g., oxidative stress, inflammation), a novel field of targeted therapeutics for AD would be identified based on these findings. Given that the behavioral manifestations are the most significant cause of caregiver burden in AD, this line of inquiry holds great promise. 

## Figures and Tables

**Table 1:** Descriptive statistics (means and standard deviations) for participants.

Alzheimer's disease	All (*N* = 272)	Male (*N* = 100)	Female (*N* = 172)
Age	77.06 (8.413)	75.70 (8.012)	77.68 (8.359)
Education	14.44 (4.758)	15.11 (3.158)	13.98 (5.802)
Dysphoria	1.78 (2.199)	1.87 (2.292)	1.76 (2.182)
Meaninglessness	.75 (1.203)	.66 (1.194)	.80 (1.173)
Apathy	1.38 (1.310)	1.49 (1.339)	1.39 (1.315)
Cognitive impairment	2.00 (1.507)	2.17 (1.569)	2.01 (1.438)
Verbal fluency	6.78 (3.109)	6.84 (3.214)	6.76 (2.954)
Boston Naming	6.19 (3.487)	5.69 (3.311)	6.59 (3.482)
Trails A	5.78 (3.027)	6.22 (2.554)	5.57 (3.243)
Trails B	5.01 (3.061)	5.77 (3.110)	4.56 (2.955)
Digit Span	8.34 (2.568)	8.85 (2.358)	8.20 (4.56)
Logical Memory I	4.11 (2.570)	4.78 (2.744)	3.85 (2.416)
Logical Memory II	4.17 (2.072)	4.72 (2.450)	3.90 (1.812)
Visual Reproduction I	4.36 (2.450)	5.29 (2.420)	4.13 (2.321)
Visual Reproduction II	5.60 (1.857)	6.12 (2.340)	5.32 (1.526)

Controls	All (*N* = 356)	Male (*N* = 114)	Female (*N* = 242)

Age	71.53 (8.566)	73.40 (8.266)	70.46 (8.559)
Education	15.64 (4.100)	16.45 (2.791)	15.17 (4.623)
Dysphoria	.74 (1.431)	.82 (1.361)	.78 (1.464)
Meaninglessness	.37 (.884)	.35 (.838)	.37 (.906)
Apathy	.96 (1.180)	.78 (1.006)	1.05 (1.245)
Cognitive impairment	1.00 (1.162)	.93 (1.119)	1.03 (1.182)
Verbal fluency	11.40 (3.005)	11.27 (3.235)	11.46 (2.095)
Boston Naming	12.11 (2.988)	13.13 (2.928)	11.63 (2.900)
Trails A	10.55 (2.636)	10.68 (2.918)	10.50 (2.500)
Trails B	11.01 (2.484)	10.90 (2.370)	11.06 (2.538)
Digit Span	11.31 (2.638)	11.64 (3.056)	11.16 (2.418)
Logical Memory I	13.01 (3.026)	12.53 (3.171)	13.23 (2.936)
Logical Memory II	13.65 (2.693)	12.96 (2.845)	13.98 (2.559)
Visual Reproduction I	12.20 (3.012)	12.25 (3.222)	12.17 (2.920)
Visual Reproduction II	13.47 (3.202)	13.57 (3.013)	13.43 (3.289)

**Table 2:** GDS subscales and neuropsychological tests for control group by gender*.

Test	Control *N* = 356								
	Males *N* = 114				Females *N* = 242				
	Standardized coefficient				Standardized coefficient				
	Scale	*β*	*T*	*P*	Scale		*β*	*T*	*P*
Verbal fluency	NS				NS	MLN	−.158	−2.541	.012
Boston Naming	NS				NS	NS			
Trails A	Apathy	−.182	−3.462	.001	NS	Apathy	−.196	−3.105	.002
Trails B	Cl	−.258	−4.989	.001	NS	CI	−.322	−5.266	.001
Digit Span	NS				NS	NS			
Logical Memory I	Cl	−.197	−3.785	.001	NS	CI	−.257	−4.118	.001
Logical Memory II	Cl	−.178	−3.395	.001	NS	CI	−.243	−3.875	.001
Visual Reproduction I	Cl	−.166	−2.929	.004	NS	CI	−.198	−2.917	.004
Visual Reproduction II	Cl	−.192	−3.401	.001	NS	CI	−.226	−3.351	.001

* Covariates entered into the equation age and education.

NS = no subscales significant; DYS = dysphoria subscale; Apathy = apathy subscale; MLN = meaninglessness subscale; Cl = cognitive impairment subscale.

**Table 3:** GDS subscales and neuropsychological tests for Alzheimer's group by gender*.

Test	AD *N* = 272								
	Males *N* = 100				Females *N* = 172				
	Standardized coefficient				Standardized coefficient				
	Scale	*β*	*T*	*P*	Scale		*β*	*T*	*P *
Verbal fluency	DYS	−.143	−2.277	.024	NS	Apathy	−.197	−2.541	.004
Boston Naming	NS				NS	NS			
Trails A	DYS	−.186	−2.791	.001	NS	Apathy	−.196	−3.105	.002
Trails B	DYS	−.279	−2.383	.018	CI		.222	2.031	.046
					DYS		−.219	−2.402	−.037
Digit Span	DYS	.131	−1.915	.050	DYS		−.329	−3.255	.002
						NS			
Logical Memory I	Apathy	−.197	−3.132	.002	NS	NS			
Logical Memory II	Cl	.183	2.953	.001	NS	CI	−.243	−3.875	.001
	Apathy	−.209	−3.356	.001					
	MLN	−.228	−3.646	.001					
Visual Reproduction I	NS				NS	Apathy	−.197	−2.589	.004
Visual Reproduction II	NS				NS	Apathy	−.200	−2.007	.048

* Covariates entered into the equation age and education.

NS = no subscales significant; DYS = dysphoria subscale; Apathy = apathy subscale; MLN = meaninglessness subscale; Cl = cognitive Impairment subscale.
